# Forecasting Explosive Bubble Regimes in Bitcoin Time Series with Multi-Step Ensemble Learning

**DOI:** 10.3390/e28101096

**Published:** 2026-10-08

**Authors:** Jian-Rong Li, Guang-Yan Zhong, Jiang-Cheng Li

**Affiliations:** School of Finance, Yunnan University of Finance and Economics, Kunming 650221, China; jianrongcareer@163.com (J.-R.L.); guangyanzhong1989@163.com (G.-Y.Z.)

**Keywords:** Bitcoin bubble, explosive regimes, time series, multi-step forecasting, ensemble learning, Model Confidence Set

## Abstract

Bitcoin bubbles exhibit locally explosive dynamics, but identifying past episodes does not establish their predictability. We combine the generalized supremum augmented Dickey–Fuller test with nine probabilistic learners and three probability-output ensembles to forecast bubble states over one-, three-, and seven-day horizons. Using daily data from January 2016 to April 2026, we identify 30 maximal uninterrupted episodes comprising 455 bubble days. Development-period validation selects the lookback window and oversampling ratios before final-test evaluation; ensemble weights are estimated from development-period rolling forecasts. No model family dominates across horizons. The constrained least-squares ensemble has the lowest final-test mean squared error at one day, whereas logistic regression has the lowest error at three and seven days. The inverse-error-weighted ensemble has the highest average precision at seven days. Moving-block bootstrap intervals overlap substantially, and the 90% Model Confidence Set retains several models at every horizon. A cross-source robustness check yields 99.58% agreement in daily bubble-state classifications. These findings support horizon-dependent probabilistic risk monitoring, while the limited number of independent episodes and low recall at longer horizons constrain the practical interpretation of the forecasts.

## 1. Introduction

Financial markets are canonical examples of complex adaptive systems in which locally explosive behavior, nonlinear state transitions, and critical phenomena emerge from the interactions of heterogeneous agents [1]. Within the critical-bubble framework, a speculative bubble can be represented as a transient super-exponential growth regime that may culminate in a critical transition [2,3,4,5]. The analogy with a physical system approaching a phase boundary provides a modeling perspective [3,4,5], linking bubble dynamics to faster-than-exponential growth and log-periodic oscillations. Related models use self-organized criticality [6] and stochastic resonance [7] to characterize financial crashes and bubbles, establishing structural analogies between extreme market states and critical phenomena in statistical physics. Related financial-risk research uses cost-sensitive boosted trees to predict banking systemic risk [8].

In this context, the Bitcoin market provides a particularly useful case for complex-system analysis. Bitcoin does not confer contractual cash flows comparable to those of stocks or bonds. Its valuation has been studied through supply and demand, use as a payment network, and models of intrinsic value [9,10,11]. These approaches do not provide a directly observed fundamental-value benchmark for the present analysis. Partida et al. investigate chaotic, self-similar, and hierarchical patterns in Bitcoin and Ethereum prices [12]. Obanya et al. use permutation entropy to study the ordinal patterns of GARCH-estimated Bitcoin volatility [13]. Liu and Tsyvinski find that cryptocurrency returns are associated with cryptocurrency-specific momentum and investor attention, rather than conventional stock-market factors [14].

Within the critical-bubble framework, positive feedback and herding can generate unsustainable, faster-than-exponential price growth associated with critical-transition risk [2]. Early rational-bubble research usually starts from the deviation of market prices from fundamental values and attempts to distinguish fundamental changes from price bubbles [15,16]. However, for assets such as Bitcoin that lack a cash-flow foundation, traditional bubble tests are also sensitive to model specification, limited samples, and periodically collapsing bubbles [17,18]. In price-bubble identification, Sornette et al. [3,4,5] explained bubbles as collective behavior near critical points and proposed the LPPL model, which uses faster-than-exponential growth and log-periodic oscillations to characterize bubble formation and potential turning points. Geuder et al. [19] examined Bitcoin bubbles using recursive explosive-root tests and an LPPL approach, while Shu and Zhu [20] used LPPLS diagnostics for real-time Bitcoin bubble-crash prediction. Phillips, Wu, and Yu [21] developed the recursive SADF approach; Phillips, Shi, and Yu [22] generalized it to GSADF to improve the detection of multiple bubbles. This generalized method was subsequently applied to cryptocurrency price-bubble studies [23,24,25]. Yao and Li compare BSADF and LPPLS methods for analyzing Bitcoin bubble bursting points [26]. Historical identification dates statistically explosive states; the forecasting question is whether recent market dynamics contain information about the occurrence or continuation of these states over a future window.

At the same time, advances in bubble identification have shaped the object of cryptocurrency prediction research. Early studies mainly relied on financial time-series models to describe conditional volatility and parameter changes. Katsiampa [27] compared the ability of different GARCH models to estimate Bitcoin volatility. Catania et al. [28] discussed cryptocurrency forecasting from the perspective of model and parameter instability. As trading variables, technical indicators, economic factors, and market uncertainty indicators have been incorporated into forecasting frameworks, machine learning methods have increasingly been used to handle nonlinear relationships and high-dimensional information structures in cryptocurrency markets [29,30,31,32]. Bagheri and Giudici evaluate Bitcoin forecasts in terms of accuracy, security against input perturbations, and explainability [33]. Multi-step forecasting is also a key issue in time-series modeling because it involves forecasting strategies, error propagation, and information extraction at different time scales, and is not a simple extension of single-step forecasting [34,35]. Related studies combine econophysics with machine learning or Bayesian methods to examine cross-market risk and forecasting performance [36,37]. AboElnasr et al. combined multifractal analysis with dictionary learning for financial-crisis risk warning, showing that complexity-related features can be transformed into learnable risk patterns [38]. Liu et al. integrated class-imbalance mitigation, adaptive ensemble learning, and interpretability into a unified financial-risk-warning framework, highlighting the joint roles of sample distribution and model complementarity in rare-risk prediction [39]. In Bitcoin bubble research, Park and Yang [40] incorporated GSADF bubble information into machine-learning models to predict market crashes following bubble formation, indicating that statistical bubble diagnostics may contain forward-looking risk information.

These studies motivate three questions for bubble-state forecasting. First, historical detection alone does not establish whether an explosive state can be forecast. Second, the crash target in Park and Yang [40] differs from the occurrence or continuation of a bubble state, whose persistence may affect performance across horizons. Third, comparisons should assess both probability accuracy and sampling uncertainty, because point-estimate rankings alone do not establish reliable model differences.

In this context, we propose a hybrid framework for multi-step bubble-state prediction that combines bubble identification, class-imbalance processing, machine learning probabilistic prediction, and model ensemble. Building on the price crash prediction study of Park and Yang [40], we shift the prediction target to the bubble state itself and the bubble process. We combine GSADF-derived statistical information with historical prices, returns, and trading volume. Our evaluation focuses on probability accuracy, ranking performance, and sampling uncertainty; it does not assess adversarial security or model explainability. We use six lookback windows and three forecast horizons to compare how different learning mechanisms capture bubbles. Drawing on the forecast-combination literature reviewed by Wang et al. [41], we construct three probability ensemble methods: equal-weighted probability ensemble, inverse-MSE-weighted probability ensemble, and constrained least-squares probability ensemble. We use these methods to examine whether bubble-warning information can be improved at the probability-output level, and we use the Model Confidence Set (MCS) of Hansen et al. [42] to compare squared-error losses while accounting for multiple candidate models.

The remainder of this paper is structured as follows. Section 2 introduces the data sources, GSADF bubble identification, prediction-label construction, machine learning models, probability ensemble methods, and MCS tests. Section 3 presents the empirical results, compares the forecast performance of the base models and ensemble methods across forecast horizons, and discusses model robustness. Section 4 summarizes the main conclusions, explains the limitations, and proposes directions for future research.

## 2. Materials and Methods

### 2.1. Research Framework

To examine whether recent Bitcoin market dynamics contain information about the near-term occurrence or continuation of statistically explosive states, and whether machine learning methods can extract such information from historical market data, this paper proposes three research questions. The first concerns bubble-state predictability, while the second and third concern model and ensemble performance:

(1) Do historical Bitcoin market dynamics contain learnable information about whether the market will enter or remain in a statistically explosive state over the next *h* days?
(2) Are there meaningful differences in the bubble-state prediction performance of machine learning models across forecast horizons?
(3) When single-model performance changes across forecast horizons, can model ensembles reduce model-selection risk and improve forecast robustness?

The research framework first uses the GSADF test to identify explosive bubble intervals in Bitcoin prices and to construct the bubble label and bubble index. Next, trading variables and bubble-state variables in historical windows are transformed into supervised learning features, and SMOTE is applied only within the training windows to address bubble sample scarcity; validation and final-test observations are never used to construct synthetic samples. Finally, nine machine learning and deep learning models are compared, three ensemble methods are constructed at the probability-output layer, and the MCS test is used to evaluate out-of-sample probabilistic prediction loss.

### 2.2. Data and Variables

This paper uses daily Bitcoin trading data as the sample. The data come from Investing.com and cover the period from 1 January 2016 to 9 April 2026. The observation window therefore extends through April 2026; the fact that the last GSADF-positive episode ends on 17 December 2024 reflects the test results rather than truncation of the dataset. For a cross-source robustness check only, we additionally use Yahoo Finance BTC/USD prices over the identical calendar sample (Appendix B). The original price data include market variables such as closing price (Price), high price (High), low price (Low), trading volume (Volume), and percentage change (ChangePct). To incorporate price-bubble identification results into the prediction model, this paper further adds the bubble label (bubble_flag) and bubble index (bubble_index) obtained from the GSADF test to the feature set. The input variables are defined in Table 1.

Table 2 reports descriptive statistics.

Let $z_{t}$ denote the original feature vector at time *t*, consisting of ${\mathrm{Price}}_{t}$, ${\mathrm{High}}_{t}$, ${\mathrm{Low}}_{t}$, ${\mathrm{Volume}}_{t}$, ${\mathrm{ChangePct}}_{t}$, $\mathrm{bubble}\text{\_}{\mathrm{flag}}_{t}$, and $\mathrm{bubble}\text{\_}{\mathrm{index}}_{t}$. For non-sequential machine learning models, we expand the historical window of length *W* into a fixed-dimensional vector:

(1) $$x_{t,W}=\mathrm{vec}\left(z_{t-W+1},z_{t-W+2},\ldots ,z_{t}\right),\;W\in \left\{7,10,14,30,60,90\right\}.$$

For the LSTM model, the time order of the variables within the window is preserved, and the input is represented as $X_{t,W}\in R^{W\times p}$. The forecast horizon is set to h∈{1,3,7} to examine the bubble state over the next 1, 3, and 7 days, respectively.

### 2.3. GSADF Bubble Identification Method

To identify periodic explosive behavior in Bitcoin prices, this study uses the Generalized Supremum Augmented Dickey–Fuller (GSADF) test proposed by Phillips et al. [22]. Compared with single-window ADF or SADF tests, GSADF allows the start and end points to roll simultaneously and is therefore more suitable for capturing price bubbles that appear and collapse multiple times over a long sample. For any recursive subsample, the ADF regression can be written as:

(2) $$\Delta {\mathrm{Price}}_{t}=\mu +\rho {\mathrm{Price}}_{t-1}+\sum_{j=1}^{k}\phi_{j}\Delta {\mathrm{Price}}_{t-j}+\varepsilon_{t},$$

${\mathrm{Price}}_{t}$ represents the Bitcoin price series, and *k* is the lag order. In the empirical test, this study uses the price-level series and sets the lag order to 0. Following Phillips et al. [22], the minimum window fraction is $r_{0}=0.01+1.8/\sqrt{T}$; with T=3752, $\lfloor Tr_{0}\rfloor =147$. The 95% critical-value sequence is obtained by Monte Carlo simulation with 1000 replications and seed 123.

Let ${\mathrm{ADF}}_{r_{1}}^{r_{2}}$ represent the subsample ADF statistic with $r_{1}$ as the starting point and $r_{2}$ as the end point. The GSADF statistic is then defined as:

(3) $$\mathrm{GSADF}\left(r_{0}\right)=\underset{\begin{array}{c} r_{2}\in \left[r_{0},1\right]\\ r_{1}\in \left[0,r_{2}-r_{0}\right] \end{array}}{\sup}{\mathrm{ADF}}_{r_{1}}^{r_{2}},$$

where $r_{0}$ is the minimum window ratio. Furthermore, the backward SADF (BSADF) statistic at time *t* is used to locate bubble intervals:

(4) $${\mathrm{BSADF}}_{t}=\underset{r_{1}\in \left[0,r_{t}-r_{0}\right]}{\sup}{\mathrm{ADF}}_{r_{1}}^{r_{t}}.$$

Given the 95% critical value ${\mathrm{CV}}_{t}$, our research defines the bubble label as:

(5) $$\mathrm{bubble}\text{\_}{\mathrm{flag}}_{t}=I\left({\mathrm{BSADF}}_{t}>{\mathrm{CV}}_{t}\right).$$

Here, I(·) is an indicator function. When ${\mathrm{BSADF}}_{t}>{\mathrm{CV}}_{t}$, it takes the value 1; otherwise, it takes the value 0. Motivated by the bubble-index approach of Park and Yang [40], we define a daily distance between the BSADF statistic and its critical value:

(6) $$\mathrm{bubble}\text{\_}{\mathrm{index}}_{t}={\mathrm{BSADF}}_{t}-{\mathrm{CV}}_{t}.$$

When $\mathrm{bubble}\text{\_}{\mathrm{index}}_{t}>0$, the BSADF statistic exceeds the corresponding critical value, indicating that the market is in a bubble state in a statistical sense. A larger value indicates a greater exceedance in test-statistic units, not a calibrated bubble probability or a measured deviation from fundamental value.

Throughout the paper, a bubble state refers to the daily indicator in Equation (5), whereas a bubble episode denotes a maximal uninterrupted run of bubble-state days. The term explosive regime is used only as a descriptive concept for the underlying locally explosive market state, not as a separate empirical counting unit.

### 2.4. Bubble Labels and Prediction Sample Construction

After completing bubble identification and bubble index construction, we proceed to the machine learning prediction stage. For any forecast horizon *h*, we define the occurrence or continuation of a bubble state within the future window as a binary response variable:

(7) $$y_{t,h}=I\left(\sum_{s=1}^{h}\mathrm{bubble}\text{\_}{\mathrm{flag}}_{t+s}>0\right),\;y_{t,h}\in \left\{0,1\right\}.$$

The model estimates the conditional probability of at least one bubble-state day within that window, given historical information:

(8) $${\hat{p}}_{t,h}=P\left(y_{t,h}=1|x_{t,W}\right).$$

Forecast origins are partitioned chronologically into a development segment and an untouched final-test segment separately for h∈{1,3,7}. To prevent future-label overlap across evaluation boundaries, the seven forecast origins immediately preceding each validation or final-test period are purged, using the maximum forecast horizon $h_{\max}=7$ as a common embargo. Within the development segment, three expanding-window validation periods are used to select the common lookback window and the model-specific SMOTE ratios. After these choices are frozen, each main specification is refitted on the corresponding purged training sample and evaluated on the final-test sample, which retains its original class distribution. The final-test sample is not used for lookback-window selection, SMOTE selection, ensemble-weight estimation, or main-model selection. Additional robustness diagnostics use the same test period as post-selection sensitivity analyses and do not feed back into the main specification.

### 2.5. SMOTE Oversampling Strategy

Because bubble samples account for a small share of the full sample, directly training a classification model may bias predictions toward the majority class. To alleviate class imbalance, we follow the SMOTE approach proposed by Chawla et al. [43]. Recent research on financial distress prediction also shows that resampling can improve the sample distribution of imbalanced training sets and enhance a model’s ability to identify minority risk events [44]. We apply synthetic oversampling only to minority-class samples in the training set. Therefore, the oversampling process does not use test-period information and does not change the out-of-sample evaluation environment. Because this paper first uses sliding windows to convert the time series into fixed-dimensional supervised-learning samples, SMOTE locally interpolates minority-class samples within the training-set window feature space rather than directly generating new future observations in the original time series. For a minority-class sample $x_{i}$, a sample $x_{\mathrm{nn}}$ is randomly selected from its *k* minority-class nearest neighbors, and a synthetic sample is generated as follows:

(9) $$x_{\mathrm{new}}=x_{i}+\lambda \left(x_{\mathrm{nn}}-x_{i}\right),\;\lambda ∼U\left(0,1\right).$$

We set k=5. Candidate SMOTE ratios range from 0.2 to 1.0 in steps of 0.1, alongside an unmodified training sample. A ratio of 0.2 targets a post-oversampling minority-class count equal to 20% of the majority-class count. If the observed ratio already meets the target, the training sample is unchanged.

**Figure A1:**
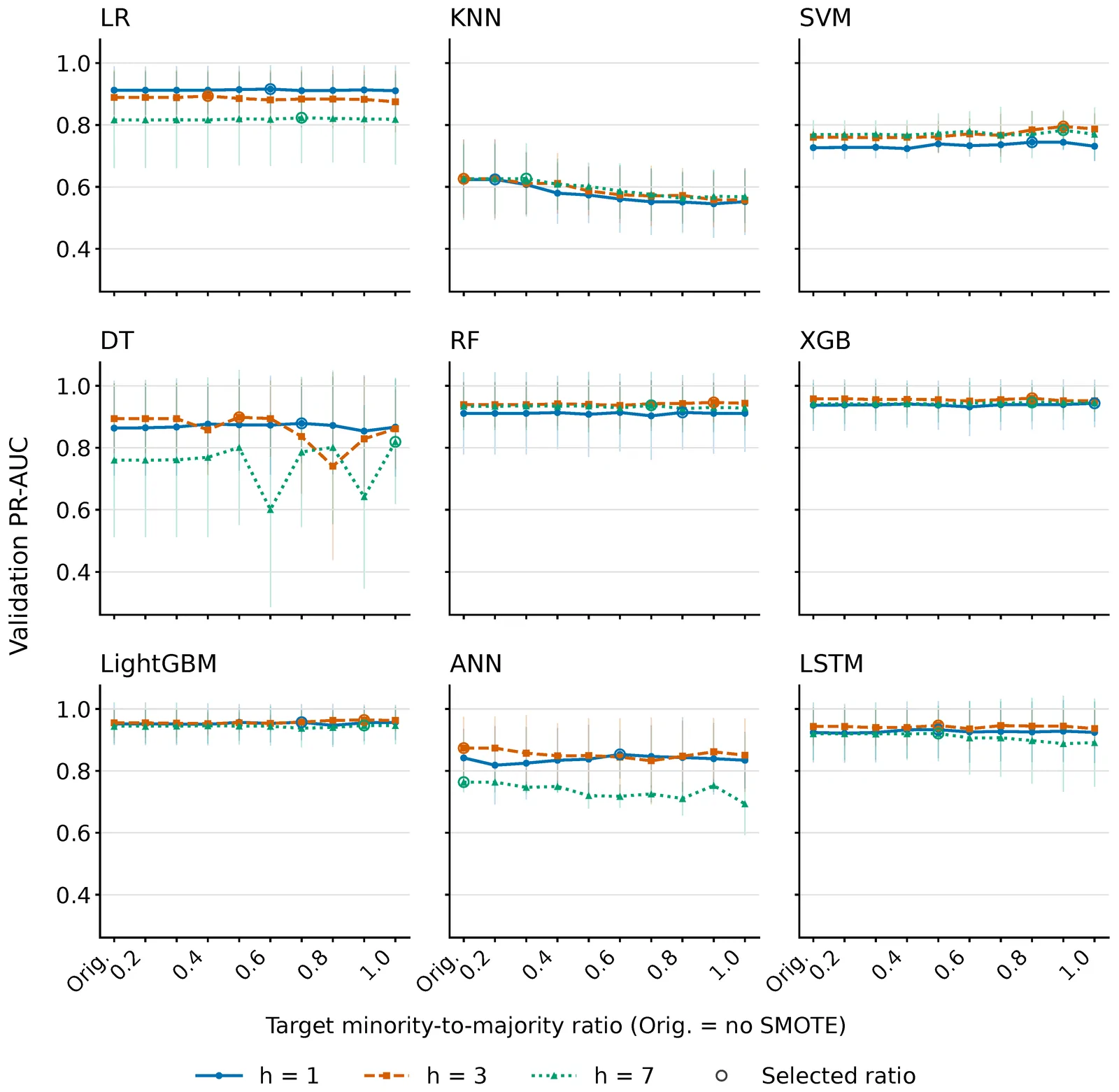
SMOTE validation PR-AUC sensitivity for nine base learners at h=1,3,7. Each panel compares the three horizons for one model. Points show means across three development-period validation samples; faint vertical bars show ±1 sample standard deviation. Small open circles in the corresponding curve color identify the selected ratios in Table 7. Orig. denotes no SMOTE. All candidate ratios are plotted; only selected horizontal-axis ticks are labeled. The lookback window is fixed at W=7 before SMOTE selection; final-test observations are excluded.

A limitation of this strategy is that SMOTE interpolates between nearest neighbors defined in feature space without regard to their temporal ordering, so a synthetic sample may combine patterns originating from different dates. For sequence models such as LSTM, the internal temporal structure of a synthesized window is therefore not guaranteed to correspond to a path that occurred historically. However, SMOTE is applied only within training windows after the temporal split; validation and final-test observations are never used to construct synthetic samples, so the out-of-sample chronology and class distribution remain intact. We additionally report development-period SMOTE-ratio sensitivity (Figure A1) and an LSTM with-versus-without-SMOTE robustness comparison (Section 3.6).

For LR, SVM, DT, and RF, the formal model specifications also retain class weighting. When a non-original SMOTE ratio is selected, resampling and class weighting are therefore used simultaneously for these models. In LR, SVM, and DT, balanced class weights are computed from the labels supplied after resampling; RF recomputes them within each bootstrap subsample. Thus, weights are not fixed at the original class frequencies. To assess how the two adjustments interact, Table A1 reports a four-way ablation—neither adjustment, SMOTE only, class weighting only, and both—while all other settings are held fixed. The ablation is used as a sensitivity analysis and does not trigger ex-post re-selection of the final specification.

### 2.6. Machine Learning and Deep Learning Models

We build machine learning models to predict the future bubble state of the Bitcoin market across different forecast horizons. Each model takes historical market characteristics as input and outputs the corresponding bubble probability. To avoid making the results depend excessively on a single algorithmic structure, this paper selects multiple machine learning and deep learning models. The main model parameter settings are shown in Table 3.

GSADF estimation was implemented in R 4.5.2 using the exuber package. Model training and evaluation were performed in Python 3.13.5 using NumPy 2.3.5, pandas 3.0.3, SciPy 1.17.0, scikit-learn 1.8.0, imbalanced-learn 0.14.1, XGBoost 3.2.0, and LightGBM 4.6.0. Neural networks were implemented in PyTorch 2.12.1+cu130, and model confidence set tests used arch 8.0.0.

#### 2.6.1. Machine Learning Models

**Logistic Regression (LR)** uses the logistic function to convert the linear combination of characteristic variables into a binary-state probability. Its basic form is
  (10) $$P\left(y_{t,h}=1|x_{t,W}\right)=\frac{1}{1+\exp[-\left(\beta_{0,h}+\beta_{h}^{⊤}x_{t,W}\right)]}.$$
  Here, $y_{t,h}=1$ indicates at least one bubble-state day within the next *h* days, and $x_{t,W}$ is the flattened historical-window feature vector defined in Equation (1). Coefficients are estimated separately for each horizon. Dichtl et al. [45] used a multivariate logistic model as a benchmark in stock market crash prediction, and Park and Yang [40] used a regularized logistic model in Bitcoin crash prediction based on bubble analysis. Because of this probabilistic classification structure, this study includes LR in the pool of bubble-state prediction models.
**K-Nearest Neighbors (KNN)** generates classification results based on the distance between the sample to be predicted and the training samples. In this paper, KNN outputs the bubble probability according to the class distribution of nearest-neighbor samples. Akyildirim et al. [46] incorporated KNN in Bitcoin futures price-direction prediction, and Ranjan et al. [47] included this method in a set of machine learning models for Bitcoin price prediction. This study therefore uses KNN as a classification model based on local proximity relationships.
**Support Vector Machine (SVM)** constructs the decision boundary by maximizing the separating margin. Considering that there may be a nonlinear relationship between the bubble state and market characteristics, this paper uses the radial basis function kernel for feature mapping, which is in the form
  (11) $$K\left(x_{i,W},x_{j,W}\right)=\exp\left(-\gamma {∥x_{i,W}-x_{j,W}∥}^{2}\right),$$
  Here, γ is the kernel parameter. Akyildirim et al. [46] applied SVM to Bitcoin futures direction prediction, and Dichtl et al. [45] applied it to stock market crash prediction. Following this line of work, this paper uses SVM as a margin-based classifier.
  **Decision Tree (DT)** forms a tree classification rule by recursively dividing the feature space. Each internal node corresponds to a feature-splitting condition, and the leaf nodes give prediction results based on the class distribution of the training samples. Ranjan et al. [47] incorporated Decision Tree into a machine learning model comparison framework for Bitcoin price prediction. This paper also follows this tree-structured classification approach in the experiments.
**Random Forest (RF)** consists of multiple decision trees, and each base tree is trained through sample and feature sampling. The final model prediction is obtained by aggregating the outputs of multiple trees. Wang et al. [31] used RF in cryptocurrency volatility prediction. Park and Yang [40] also incorporated RF into a Bitcoin crash prediction framework based on GSADF bubble analysis. Accordingly, this study uses RF as one of the tree-ensemble models.
**Extreme Gradient Boosting (XGBoost)** builds an additive tree model based on the gradient boosting framework, and corrects the previous round of prediction errors by gradually adding new weak learners. The general form can be expressed as
  (12) $$F_{t,h}=b_{h}+\sum_{m=1}^{M}f_{m,h}\left(x_{t,W}\right),\;{\hat{p}}_{t,h}=\sigma \left(F_{t,h}\right),$$
  Here, $F_{t,h}$ is the raw score, $b_{h}$ is the initial score, $f_{m,h}\left(\cdot \right)$ is the contribution of tree *m* including its learning-rate scaling, and $\sigma \left(z\right)=1/\left(1+e^{-z}\right)$ maps the score to a probability. Ranjan et al. [47] compared the Bitcoin prediction performance of machine learning models such as XGBoost under different sample dimensions. Bagheri and Giudici [33] also incorporated XGBoost into a Bitcoin prediction model system. Similarly, this paper uses XGBoost to represent the gradient-boosting tree structure in bubble-state prediction.
**Light Gradient Boosting Machine (LightGBM)** is also a gradient-boosting tree model. Its training process usually combines a histogram-based splitting strategy with leaf-wise growth. Dudek et al. [48] built deterministic and probabilistic forecasting frameworks for Bitcoin volatility using LightGBM. We therefore include LightGBM as a gradient-boosting model for cryptocurrency prediction.

#### 2.6.2. Deep Learning Models

**Artificial Neural Network (ANN)** consists of an input layer, two hidden layers, and an output layer, and the input features are transformed layer by layer through weighted connections and nonlinear activation functions. For notational simplicity, given $x_{t,W}$, a generic hidden-layer transformation is $a_{t,h}=f\left(A_{h}x_{t,W}+b_{h}\right)$, where $A_{h}$ and $b_{h}$ are the weight matrix and bias vector, and f(·) is the activation function. Compared with linear classification models, ANN can capture potentially complex relationships among technical indicators, price changes, and bubble states through multi-layer nonlinear mapping. This paper adopts a feedforward neural network structure and uses the sigmoid function in the output layer to convert the model output into a bubble probability, ${\hat{p}}_{t,h}=\sigma \left(v_{h}^{⊤}a_{t,h}+b_{o,h}\right)$. Park and Yang [40] used an ANN model in Bitcoin crash prediction based on bubble analysis, so our study also incorporates ANN as a neural-network base learner to test the effectiveness of nonlinear feature representation in bubble-state prediction.
**Long Short-Term Memory (LSTM) network** is a recurrent neural network model that updates the cell state and hidden state through input, forget, and output gates. Whereas ANN receives the flattened historical window, LSTM processes the rows $z_{s}$ of $X_{t,W}$ in temporal order, for s=t−W+1,…,t. At each step it combines $z_{s}$ with the previous hidden state $a_{s-1}$ through gate control. The recurrence below is within a window; horizon-specific network parameters are suppressed for readability. Its core update process can be summarized as
  (13) $$c_{s}=f_{s}⊙c_{s-1}+i_{s}⊙{\tilde{c}}_{s},$$
  Here, $f_{s}$ and $i_{s}$ are the forget and input gates, $c_{s}$ is the cell state, ${\tilde{c}}_{s}$ is the candidate cell-state vector computed from the current input and previous hidden state, and ⊙ denotes element-wise multiplication. This structure enables the model to capture time-dependent characteristics such as price momentum, volatility clustering, and bubble-state persistence within a historical window. LSTM maps the final hidden state to ${\hat{p}}_{t,h}$, the probability of at least one bubble-state day in the future window. Wang et al. [31] used LSTM in cryptocurrency volatility prediction, and Park and Yang [40] also used LSTM for Bitcoin crash prediction based on GSADF bubble information. This paper therefore uses LSTM to describe the sequential structure of historical market states.

Table 3 lists the main model parameters. All models use the same temporal splits. LSTM receives a sequence of historical feature vectors; the other models receive the same history as a flattened vector. The class-weight settings for LR, SVM, DT, and RF remain active when SMOTE is selected. Their separate and joint effects are examined in Table A1.

### 2.7. Model Ensemble Methods

A single model may change its ranking across forecast horizons and evaluation metrics. To reduce dependence on any specific model, this paper constructs three probability ensemble methods: an equal-weighted probability ensemble, an inverse-MSE-weighted probability ensemble, and a constrained least-squares probability ensemble. These methods build on the probability-output ensemble methods reviewed by Wang et al. [41] and examine whether ensemble learning at the probability-output layer can improve bubble-probability prediction. Assume that the bubble-probability prediction of sample *i* by the *m*th benchmark model is ${\hat{p}}_{i,h,m}$, the number of benchmark models is *M*, and the true label is $y_{i,h}\in \left\{0,1\right\}$. Here, *i* indexes forecast origins within a given sample; $y_{i,h}$ and ${\hat{p}}_{i,h,m}$ correspond to $y_{t,h}$ and the model-specific forecast at that origin. All three ensemble methods can be written in a unified form:

(14) $$\begin{array}{cc} {\hat{p}}_{i,h}^{\mathrm{ens}}\; & =\sum_{m=1}^{M}w_{m,h}{\hat{p}}_{i,h,m}, \end{array}$$

(15) $$\begin{array}{cc} w_{m,h} & \geq 0,\;\sum_{m=1}^{M}w_{m,h}=1. \end{array}$$

Here, $w_{m,h}$ denotes the contribution of base model *m*. The three ensembles differ in how their weights are estimated. We estimate a separate weight vector for each horizon and hold it fixed throughout the final test; performance gains from combination are assessed empirically rather than assumed.

Ensemble-weight estimation uses 69 chronological monthly rolling-origin blocks per horizon, from August 2017 through April 2023, within the development sample. At each origin, the model is fitted on an expanding history after purging the seven forecast origins immediately before the evaluation block. The final April block ends on 18, 17, and 14 April for h=1,3,7, respectively. The selected W=7 and model-specific SMOTE settings are fixed before generating these development predictions. This produces $n_{c,h}=2087$, 2086, and 2083 probabilities for the respective horizons. These are rolling pseudo-out-of-sample predictions conditional on the development-selected settings, not predictions from randomly shuffled cross-validation folds. No final-test observations enter weight estimation. Let ${\hat{p}}_{i,h,m}^{c}$ denote the resulting probability from model *m* for development observation *i*. Then

(16) $$e_{m,h}=\frac{1}{n_{c,h}}\sum_{i=1}^{n_{c,h}}{\left(y_{i,h}-{\hat{p}}_{i,h,m}^{c}\right)}^{2}.$$

Here, $n_{c,h}$ counts the pooled development evaluation observations. Equation (16) weights each observation equally, whereas the monthly stability summary gives equal weight to each monthly block. The same pooled probability matrix is used in Equation (21).

#### 2.7.1. Equally Weighted Model Ensemble (EW-ME)

The equal-weighted model ensemble assigns the same weight to all base models:

(17) $$\begin{array}{cc} w_{m,h}^{\mathrm{EW}} & =\frac{1}{M}, \end{array}$$

(18) $$\begin{array}{cc} {\hat{p}}_{i,h}^{\mathrm{EW}} & =\frac{1}{M}\sum_{m=1}^{M}{\hat{p}}_{i,h,m}. \end{array}$$

The equal-weighted method does not estimate additional parameters, and its economic implication is that each model is regarded as an equally reliable source of information; when it is impossible to confirm which model is dominant in the long term, the simple average can disperse single-model errors.

#### 2.7.2. Inverse Mean Squared Error Weighted Model Ensemble (IMSE-ME)

The inverse mean squared error weighted model ensemble assigns weights based on rolling development errors:

(19) $$\begin{array}{cc} w_{m,h}^{\mathrm{IM}} & =\frac{e_{m,h}^{-1}}{\sum_{j=1}^{M}e_{j,h}^{-1}}, \end{array}$$

(20) $$\begin{array}{cc} {\hat{p}}_{i,h}^{\mathrm{IM}} & =\sum_{m=1}^{M}w_{m,h}^{\mathrm{IM}}{\hat{p}}_{i,h,m}. \end{array}$$

The smaller the error and the larger $e_{m,h}^{-1}$, the higher the weight assigned to the model. The method gives more weight to models with better development-period probability accuracy while keeping all model weights non-negative and summing to 1.

#### 2.7.3. Constrained Least-Squares Model Ensemble (CLS-ME)

Constrained least-squares model ensemble does not rely on individual model errors alone. Instead, it directly searches for a set of joint weights that minimizes the MSE of the rolling development ensemble probabilities:

(21) $$\begin{array}{cc} w_{h}^{\mathrm{CLS}} & =\arg\min_{w_{h}}\sum_{i=1}^{n_{c,h}}{\left(y_{i,h}-\sum_{m=1}^{M}w_{m,h}{\hat{p}}_{i,h,m}^{c}\right)}^{2},\\ s.t.\; & w_{m,h}\geq 0,\;m=1,\ldots ,M,\;\sum_{m=1}^{M}w_{m,h}=1. \end{array}$$

This setting allows the weights to reflect the complementary relationship between the models: if the error directions of two models are similar, the benefits of assigning them high weights simultaneously are limited; if a model can compensate for the prediction blind spots of other models, it may still gain weight in the joint optimization even when its individual MSE is not the lowest. Therefore, the constrained least-squares model ensemble places more emphasis on the overall probability error after combination.

### 2.8. Model Confidence Set (MCS) Procedure

The Model Confidence Set (MCS) test is used to compare prediction loss differences across multiple candidate models [42]. Following Yang et al. [49], we combine MCS with machine learning in a hybrid prediction framework for high-dimensional and imbalanced systems. This study includes nine base models and three ensemble methods in the candidate set $M_{0}$ and uses test-set MSE loss as the comparison criterion. The following comparison is conducted separately for each horizon *h*. For the probability prediction ${\hat{p}}_{t,h}^{\left(i\right)}$ of model *i* at time *t*, the single-period loss is defined as

(22) $$L_{i,t,h}={\left(y_{t,h}-{\hat{p}}_{t,h}^{\left(i\right)}\right)}^{2}.$$

The lower the loss, the closer the bubble probability given by the model is to the true state.

Let $T_{h}$ be the number of final-test forecast origins for horizon *h*, indexed by *t* in the following sums. The loss difference between any two models *i* and *j* is

(23) $$\begin{array}{cc} d_{ij,t,h}\; & =L_{i,t,h}-L_{j,t,h}, \end{array}$$

(24) $$\begin{array}{cc} {\bar{d}}_{ij,h}\; & =\frac{1}{T_{h}}\sum_{t=1}^{T_{h}}d_{ij,t,h}. \end{array}$$

If ${\bar{d}}_{ij,h}>0$, the average loss of model *i* is higher than that of model *j*; a negative value indicates lower loss, and zero indicates equal average loss.

The core null hypothesis of the MCS test is that all models in the candidate set have equivalent predictive capabilities:

(25) $$H_{0,M}:E\left(d_{ij,t,h}\right)=0,\;\forall i,j\in M.$$

If this null hypothesis is rejected, the MCS procedure progressively eliminates models with relatively poor losses and continues testing on the remaining set until equal predictive ability cannot be rejected. The MCS procedure is implemented at the 90% confidence level (α=0.10). Following Hansen et al. [42], model *i* is included in the 90% model confidence set when its MCS *p*-value satisfies ${\hat{p}}_{i,h}^{\mathrm{MCS}}\geq \alpha$. Accordingly, the superior model set is defined as

(26) $${\hat{M}}_{0.90}^{*}=\left\{i\in M_{0}:{\hat{p}}_{i,h}^{\mathrm{MCS}}\geq 0.10\right\}.$$

The resulting set contains the models whose predictive losses cannot be shown to be significantly inferior under the selected MSE loss criterion.

In implementation, the 90% MCS uses 1000 stationary-bootstrap replications, an expected block size of 10, method R, and seed 42. MCS *p*-values are interpreted only through membership in the conventional 90% model confidence set.

### 2.9. Evaluation Metrics

We define TP, FP, TN, and FN as true positives, false positives, true negatives, and false negatives, respectively. To evaluate model performance in bubble-state prediction, this study uses confusion-matrix classification metrics such as Accuracy, Precision, Recall, and F1, as analyzed by Sokolova and Lapalme [50]. Following the precision–recall evaluation perspective of Davis and Goadrich [51], we assess minority-class ranking using non-interpolated average precision, reported below as PR-AUC. For binary probability forecasts, MSE is the binary Brier score [52].

**Precision** measures the proportion of real bubble samples among all samples predicted as bubbles, reflecting the accuracy of bubble warning signals:
  (27) $$\mathrm{Precision}=\frac{\mathrm{TP}}{\mathrm{TP}+\mathrm{FP}}.$$
**Recall** measures the proportion of all real bubble samples that are correctly identified as bubbles by the model, reflecting the extent to which the model misses bubble events:
  (28) $$\mathrm{Recall}=\frac{\mathrm{TP}}{\mathrm{TP}+\mathrm{FN}}.$$
**F1** is the harmonic mean of Precision and Recall, used to comprehensively measure warning accuracy and bubble capture ability:
  (29) $$F1=\frac{2\times \mathrm{Precision}\times \mathrm{Recall}}{\mathrm{Precision}+\mathrm{Recall}}.$$
**Accuracy** measures the proportion of correctly classified samples among all samples. Due to the low proportion of bubble samples, this indicator is retained as a reference definition; the main results emphasize Precision, Recall, F1, PR-AUC, and probability MSE:
  (30) $$\mathrm{Accuracy}=\frac{\mathrm{TP}+\mathrm{TN}}{\mathrm{TP}+\mathrm{TN}+\mathrm{FP}+\mathrm{FN}}.$$

PR-AUC is reported as non-interpolated average precision, which summarizes minority-class ranking performance. For recall levels $R_{k}$ and precision values $P_{k}$ at successive score thresholds, ordered by increasing recall with $R_{0}=0$,

(31) $$\mathrm{PR}\text{-}\mathrm{AUC}=\sum_{k=1}^{K}\left(R_{k}-R_{k-1}\right)P_{k}.$$

This definition uses recall increments as weights and does not apply trapezoidal interpolation.

**MSE** measures the mean squared error between the predicted probability and the true binary label, used to evaluate overall probabilistic prediction accuracy (the Brier score for binary outcomes), rather than calibration alone:
  (32) $$\mathrm{MSE}=\frac{1}{n}\sum_{i=1}^{n}{\left(y_{i,h}-{\hat{p}}_{i,h}\right)}^{2}.$$

Classification metrics use a fixed probability threshold of 0.5. To contextualize MSE under class imbalance, a Naive-0 benchmark assigns ${\hat{p}}_{i,h}=0$ to every final-test observation. Its MSE equals the positive-class share of the corresponding horizon-specific test sample.

### 2.10. Uncertainty Assessment

To quantify uncertainty in the final-test point estimates, we construct 95% percentile confidence intervals for MSE and PR-AUC using 1000 paired moving-block bootstrap replications with block length 10. For a recent application of moving-block bootstrap to forecast-accuracy inference, see Doko Tchatoka and Haque [53]. This length exceeds the maximum forecast horizon $h_{\max}=7$ and preserves blocks of short-range serial dependence associated with overlapping multi-step labels. Each bootstrap draw resamples the true label jointly with the frozen probability outputs of all 12 models so that cross-model dependence is preserved. The resulting intervals are conditional on the fitted predictions and quantify final-test sampling uncertainty; they do not incorporate model-training or model-selection uncertainty.

## 3. Results and Discussion

### 3.1. Bitcoin Market Bubble Identification Results

#### 3.1.1. Bitcoin Market Bubble Episodes

This study uses the GSADF test to identify explosive behavior in Bitcoin prices between January 2016 and April 2026. The GSADF statistic is 10.6858, exceeding the corresponding Monte Carlo 95% critical value of 2.5139, so the null hypothesis of no explosive episode is rejected at the 5% level. The full-sample test rejects the absence of explosive behavior; the daily BSADF rule below dates the individual episodes.

**Figure 1:**
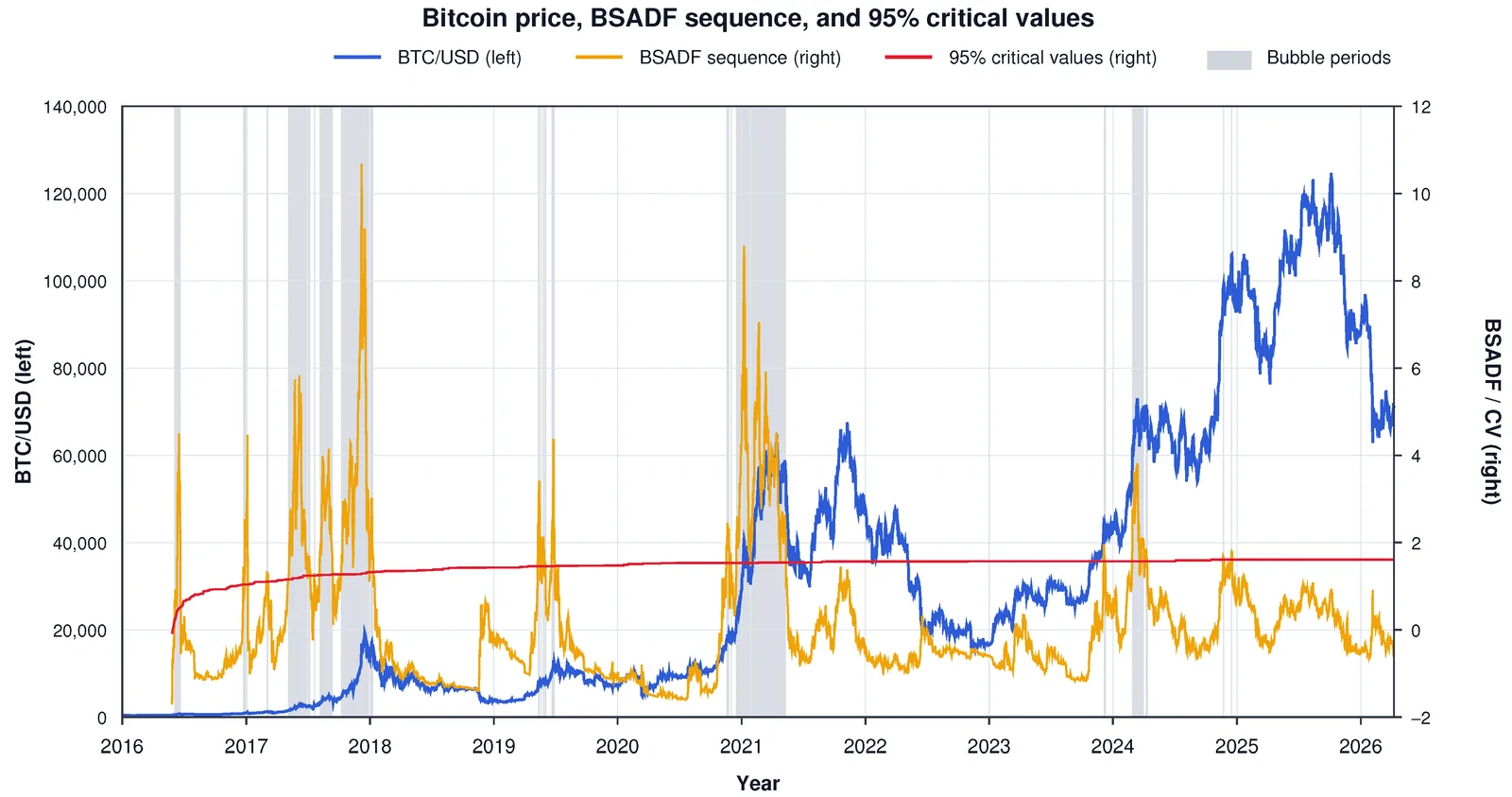
Bitcoin price, BSADF statistic, 95% critical value and bubble periods. Shading identifies 30 maximal uninterrupted episodes (455 days), with the last bubble day on 17 December 2024 in a sample ending on 9 April 2026. Very short intervals use a minimum display width; Table 4 gives their exact dates.

The shaded areas in Figure 1 show all statistically identified bubble episodes. Across the full sample, the daily BSADF rule identifies 30 maximal uninterrupted bubble episodes comprising 455 bubble days, with episode duration ranging from 1 to 104 days. Several long episodes are concentrated in 2017 and 2020–2021, whereas the nine episodes detected from December 2023 onward last only 1–21 days. The recent pattern is therefore descriptively more fragmented and pulse-like, although this should not be interpreted as a formally tested structural break. Because contiguous bubble days within an episode are not independent, the effective event count remains far smaller than the number of daily observations; uncertainty in the subsequent forecast metrics is therefore assessed using moving-block bootstrap intervals.

In Figure 1, the blue line (left axis) represents the daily closing price of Bitcoin against the US dollar; the orange line (right axis) represents the BSADF statistic calculated using the rolling window; the red curve represents the critical value at the 95% significance level; and the gray shaded areas represent the identified price-bubble intervals.

#### 3.1.2. Bitcoin Price Bubble Evidence

The BSADF rule dates intervals of statistically explosive price behavior. Table 4 reports all maximal uninterrupted episodes; it applies no minimum-duration filter and does not merge gaps.

Table 4 reports all identified price-bubble episodes within the sample, including their start and end dates, duration, mean bubble index, and cumulative price change. These daily bubble-state variables are then incorporated into the multi-step forecasting framework for h=1,3,7.

To assess whether bubble dating is specific to the primary data provider, we repeat the GSADF procedure using Yahoo Finance BTC/USD prices over the identical 1 January 2016–9 April 2026 sample while holding lag = 0, the minimum window of 147, the price-level specification, and the 95% critical-value sequence fixed. Yahoo Finance identifies 454 bubble days and 30 episodes, compared with 455 days and 30 episodes in the Investing.com data. Daily bubble-state agreement is 99.58%, the Jaccard similarity of identified bubble days is 0.9675, and the Pearson correlation between the two BSADF sequences is 0.9983. The main explosive periods are therefore highly stable across the two providers, with remaining differences concentrated around a small number of short or boundary dates (Appendix B).

### 3.2. Machine Learning Prediction Experiment Design

#### 3.2.1. Sliding Window

Our study uses sliding time windows to construct supervised-learning samples. Specifically, six lookback windows of 7, 10, 14, 30, 60, and 90 days are set, and price, trading volume, and bubble-state characteristics from the past window are extracted as inputs. Three forecast horizons of 1, 3, and 7 days are then used to predict whether at least one day in the future window is in a bubble state. This design yields 18 forecast settings.

#### 3.2.2. Development and Final-Test Partitioning

**Figure 2:**
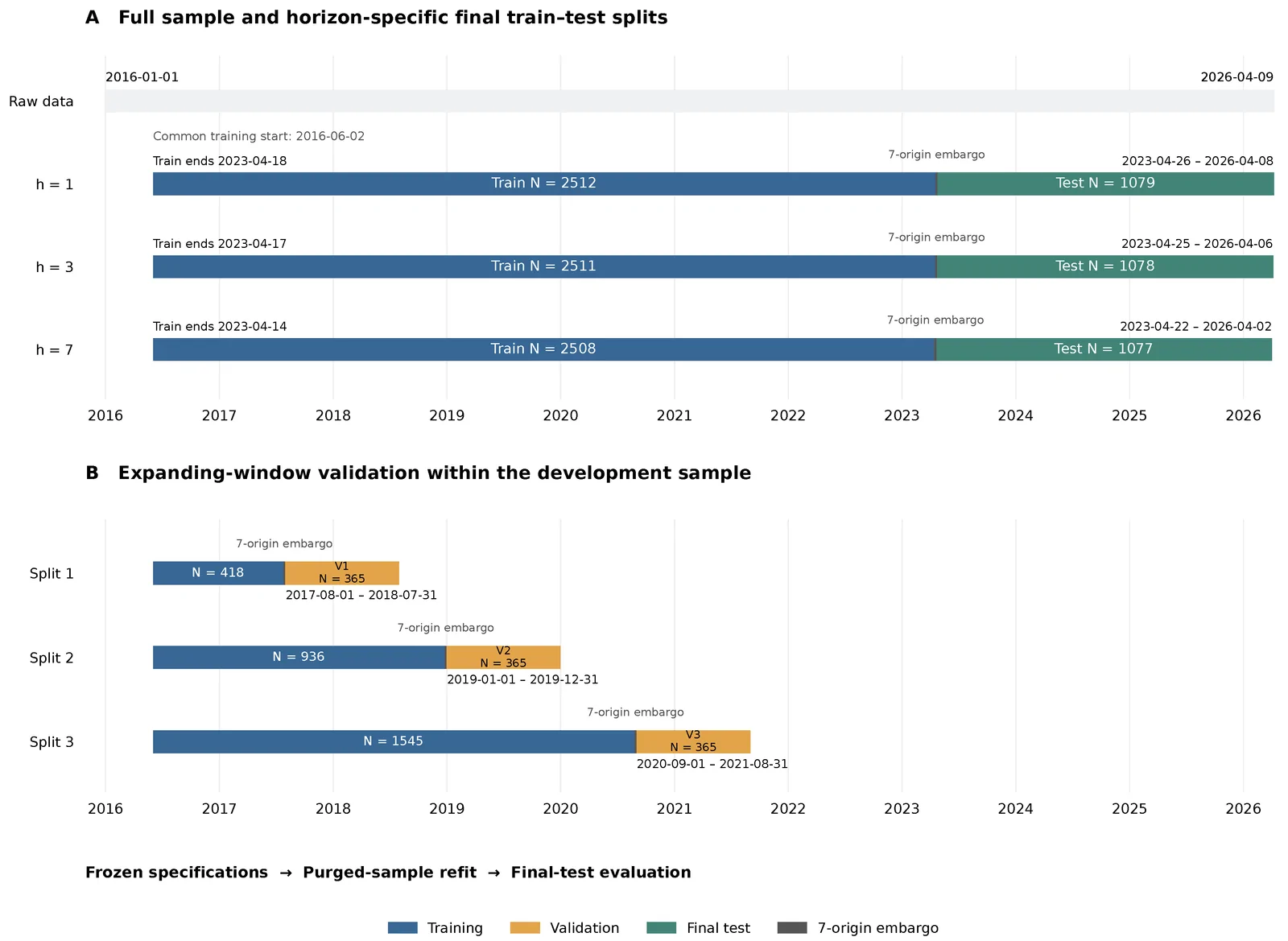
Sample partitioning and expanding-window validation design.

For each forecast horizon, forecast origins are partitioned chronologically into a development sample and an untouched final-test sample, with a common seven-origin embargo immediately before the final test. The initialized daily bubble flag is positive on 12.62% of days, but the horizon-specific prediction labels and class shares differ across *h* and across the development/final-test subsets. Table 5 reports the resulting sample composition, and Figure 2 summarizes the development-validation-final-test design.

Figure 2 presents the sample partitioning and validation design.

#### 3.2.3. Oversampling Design

Candidate SMOTE ratios from 0.2 to 1.0 are evaluated only within the development sample. The common lookback window is selected first using the validation design in Section 3.2.4; after W=7 is frozen, the SMOTE ratio is selected separately for each model and forecast horizon using mean validation PR-AUC across the three development periods.

#### 3.2.4. Sliding Window Comparison

To preserve cross-model comparability, a single common lookback window is selected before final-test evaluation using development-period validation only. For each candidate W∈{7,10,14,30,60,90}, PR-AUC is first averaged across the three held-out validation periods for each model-horizon pair and then summarized across the resulting 27 pairs, using the original class distribution without SMOTE. Table 6 shows that W=7 attains the highest mean validation PR-AUC (0.8521), followed by W=10 (0.8340). Accordingly, W=7 is frozen for all subsequent SMOTE selection, ensemble-weight estimation, model refitting, and final-test evaluation. The final test sample is not used at any stage of window selection.

The window and SMOTE comparisons are treated as development-stage tuning choices rather than as families of final-test hypothesis tests. Accordingly, no *p*-values or superiority claims are attached to validation PR-AUC differences; the SMOTE sensitivity profiles are reported in Appendix A. The final-test sample is not used for tuning or main-model selection, while formal multiple-model inference on final-test MSE is conducted using the MCS procedure in Section 3.5.

#### 3.2.5. Oversampling Ratio Sensitivity Analysis

Figure A1 reports development-period validation PR-AUC across candidate SMOTE ratios after W=7 has been frozen. The response is model- and horizon-specific rather than uniformly hump-shaped. The curves and their standard deviations describe variation across only three validation periods; they are not tests of statistically significant differences. Some model-horizon pairs are selected without oversampling. Consequently, a common SMOTE ratio is not imposed. Table 7 reports the model- and horizon-specific ratio that maximizes mean validation PR-AUC across the three development periods. These choices are frozen before final-test evaluation.

#### 3.2.6. Development-Period Base-Model Performance

Table 8 summarizes rolling pseudo-out-of-sample performance within the development sample. These development-period predictions are used to estimate ensemble weights before final-test evaluation.

Mean monthly MSE increases with the horizon for every base model, as does its temporal standard deviation. XGB has the lowest mean at h=1 and h=3, whereas RF has the lowest mean at h=7. The standard deviations describe variation across 69 monthly development blocks, not standard errors. These development-period summaries characterize base-model stability and supply the prediction losses used in ensemble-weight estimation.

### 3.3. Ensemble Construction and Development-Period Weights

**Figure 3:**
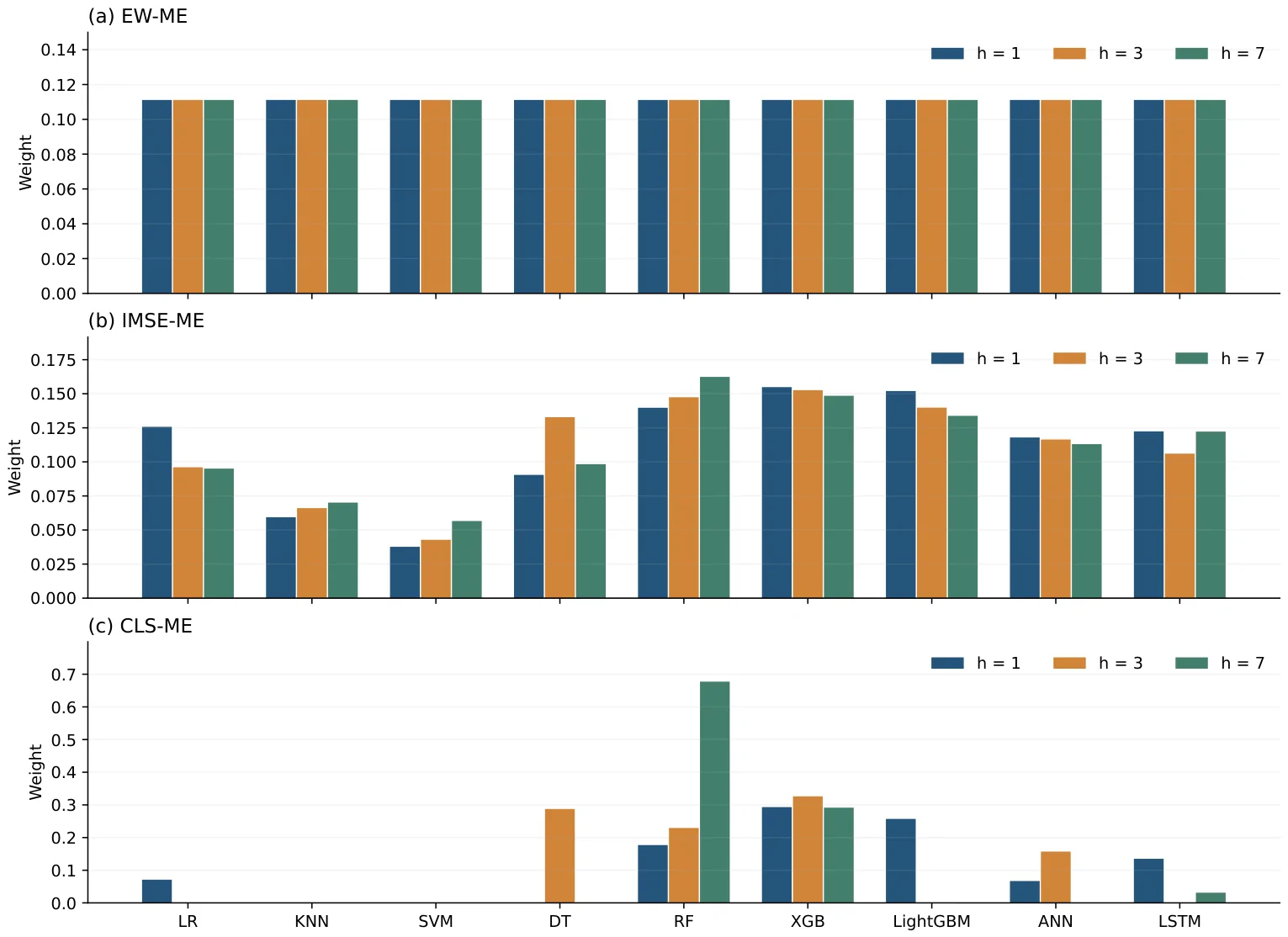
Model weights of three ensemble methods across forecast horizons. Weights use development-period rolling out-of-sample probabilities with W=7 and a seven-origin purge. Final-test observations are excluded from weight estimation.

Based on the rolling development-period predictions of the nine base learners, we construct three probability-output ensembles. Figure 3 reports the horizon-specific weights estimated exclusively from the development sample. These weights are frozen before final-test evaluation of the main specifications.

EW-ME assigns weight 1/9 to every base learner. IMSE-ME weights each learner by its inverse pooled development MSE, while CLS-ME jointly minimizes the pooled development squared error subject to non-negative weights summing to one. These weights remain fixed throughout final-test evaluation. A larger weight describes a contribution to this fitted combination; it does not establish standalone superiority.

### 3.4. Final-Test Comparative Performance

After all base-model specifications and ensemble weights have been frozen, the nine base learners and three ensembles are evaluated on the final-test sample. Table 9 reports their performance together with the Naive-0 benchmark.

On the untouched final test (Table 9), the lowest MSE at h=1 is obtained by CLS-ME (0.0125), with IMSE-ME (0.0126) and EW-ME (0.0128) close behind. At h=3 and h=7, LR attains the lowest MSE, at 0.0196 and 0.0311, respectively. PR-AUC yields a different ranking: RF is highest at h=1 (0.8730), LightGBM at h=3 (0.8601), and IMSE-ME at h=7 (0.8226). All 12 fitted models have lower MSE than the horizon-specific Naive-0 benchmark, whose MSE equals the positive-class share of the corresponding final-test sample. These results show that probability accuracy and minority-class ranking do not identify a single universally best model.

Table 10 reports the corresponding confidence intervals.

The moving-block bootstrap intervals in Table 10 are relatively wide and overlap substantially for many models, particularly at longer horizons. Thus, small differences in final-test point estimates should not be interpreted as precise or definitive model separation. The intervals quantify uncertainty conditional on the frozen predictions and complement, rather than replace, the multiple-model loss comparison conducted with the MCS procedure.

Table 9 shows that the benefit of probability ensembling is horizon-dependent rather than universal. At h=1, the three ensemble methods occupy the three lowest MSE positions, indicating a short-horizon probability-accuracy advantage from probability combination. At h=3 and h=7, however, LR has the lowest MSE, although the ensemble methods remain competitive; at h=7, IMSE-ME also attains the highest PR-AUC. Accordingly, the ensemble results are interpreted as evidence of horizon-dependent complementarity rather than uniform dominance. The following MCS analysis assesses whether the observed final-test MSE differences are statistically distinguishable within the 12-model candidate set.

### 3.5. MCS Test Results

Table 11 compares the 12 models using squared-error loss and the conventional 90% MCS.

Table 11 reports the conventional 90% MCS results using squared-error loss. At h=1, SVM and DT are excluded from the 90% model confidence set, while the remaining ten models are retained; CLS-ME has the lowest point-estimate MSE. At h=3, only DT is excluded, and LR has the lowest point-estimate MSE. At h=7, all 12 models remain in the 90% model confidence set, while LR has the lowest point-estimate MSE. The MCS therefore provides limited separation among the better-performing models and does not support a claim that a single model family or ensemble rule is uniformly superior across forecast horizons. Retention in the MCS indicates that a model cannot be shown to be inferior under the specified loss and confidence level; it should not be interpreted as proof of equivalence or superiority.

### 3.6. Robustness Checks

Table 12 compares LSTM with and without SMOTE while holding its other settings fixed.

Table 12 shows that the effect of SMOTE on LSTM is horizon-dependent rather than uniformly beneficial or harmful. At h=1, removing SMOTE improves MSE, PR-AUC, Recall, Precision, and F1. At h=3 and h=7, the no-SMOTE specification has higher PR-AUC, whereas the SMOTE specification has slightly lower MSE and higher Recall and F1. These comparisons do not isolate temporal distortion as the cause of weak recall; rather, SMOTE changes the trade-off between ranking performance and threshold-based detection across horizons.

The class-weight ablation in Table A1 shows that using SMOTE and class weighting together is not uniformly superior to either adjustment alone or neither adjustment. Combined correction changes the trade-off between probability error and minority-class detection. This comparison cannot, by itself, identify over-correction as the causal mechanism. The main specification remains fixed, and these final-test diagnostics are not used to select a replacement model.

## 4. Conclusions

This study examines whether the bubble state of the Bitcoin market can be predicted in advance and constructs a multi-step prediction framework that couples GSADF-based explosive-regime identification with class-imbalance processing, machine learning models, and probability-level model ensemble. In contrast to the crash-prediction target of Park and Yang [40], our target is the occurrence or continuation of a statistically explosive state within a future window.

Empirically, the GSADF procedure identifies 30 maximal uninterrupted bubble episodes comprising 455 bubble days between 2016 and 2026. Episode duration ranges from 1 to 104 days, and the episodes detected from December 2023 onward are shorter and more fragmented than several of the longer episodes observed in 2017 and 2020–2021. A cross-source robustness check using Yahoo Finance produces 454 bubble days and 30 episodes, with 99.58% daily state agreement and a bubble-day Jaccard similarity of 0.9675, indicating that the main explosive-period dating is highly stable to the choice of these two data providers.

For bubble-state forecasting, the design separates development-period tuning from final-test evaluation of the frozen main specifications. Development validation selects a common lookback window of W=7, after which SMOTE ratios are chosen separately by model and forecast horizon. The validation sensitivity analysis does not support a single universally optimal oversampling ratio, and the LSTM and class-weight ablations show that imbalance correction involves horizon- and model-specific trade-offs rather than an automatic performance gain.

Final-test results also show clear horizon dependence. CLS-ME has the lowest MSE at h=1, whereas LR has the lowest MSE at h=3 and h=7. PR-AUC rankings differ: RF is highest at h=1, LightGBM at h=3, and IMSE-ME at h=7. All 12 fitted models have lower MSE than the corresponding Naive-0 benchmark, but the moving-block bootstrap intervals are wide and substantially overlapping for many models. Consistently, the conventional 90% MCS retains multiple models at every horizon and retains all 12 models at h=7. These findings do not support universal ensemble superiority; instead, they indicate that ensemble methods are particularly competitive for short-horizon probability accuracy and remain useful as a way to reduce dependence on a single learner when rankings vary across horizons.

In terms of research significance, this study links statistical detection of locally explosive Bitcoin states with multi-step probabilistic forecasting. The resulting probabilities should be interpreted as complementary risk-monitoring information rather than as trading signals. Practical usefulness becomes more limited as the forecast horizon lengthens: at h=7, the highest final-test Recall is 0.5065, so approximately half of realized positive labels remain undetected even by the best recall-performing model. The framework has also not been evaluated in terms of transaction costs or economic utility.

Several limitations remain. Although 30 episodes are identified, many are short and temporally clustered, so the effective number of independent events is still much smaller than the number of daily observations. The reported bootstrap intervals are conditional on frozen predictions and do not incorporate model-training or selection uncertainty. MCS inference is horizon-specific and does not provide simultaneous coverage across all horizons or the full tuning search. The target combines new bubble-state days with continuation of existing states; the results therefore do not isolate the ability to forecast bubble onset. The forecasting features are mainly technical trading variables and GSADF-derived states; on-chain, derivatives-market, macro-financial, and sentiment information are not included. Cross-source robustness is assessed using two providers and therefore does not establish invariance across all exchanges or data vendors. Finally, the analysis identifies explosiveness statistically but does not estimate dynamical and complexity measures such as Lyapunov exponents, permutation entropy, or fractal dimensions. Future research can extend the framework to richer information sets, broader cross-market validation, and rolling real-time evaluation, while distinguishing bubble formation, continuation, and exit stages.

## Figures and Tables

**Table 1:** Input variable definitions.

Variable Name	Column Name	Definition
Closing price	Price	Bitcoin closing price for the day (USD)
Highest price	High	Bitcoin’s highest transaction price of the day (USD)
Lowest price	Low	Bitcoin’s lowest transaction price of the day (USD)
Volume	Volume	Bitcoin trading volume for the day
Percentage change	ChangePct	Daily percentage change in the Bitcoin price (%)
Bubble label	bubble_flag	1 indicates a bubble state, and 0 indicates a non-bubble state
Bubble index	bubble_index	A larger value indicates a stronger bubble signal

**Table 2:** Descriptive statistics of input variables.

Variable	*N*	Mean	Std. dev.	Skewness	Excess Kurtosis
Price	3752	31,462.8983	32,518.8854	1.0945	0.1284
High	3752	32,087.8873	33,052.9497	1.0814	0.0892
Low	3752	30,755.4362	31,938.3928	1.1111	0.1785
Volume	3752	16,462,450.5330	178,602,904.8062	16.1143	297.1574
Change (%)	3752	0.1990	3.5306	−0.1103	8.3024
bubble_flag	3605	0.1262	0.3321	2.2521	3.0734
bubble_index	3605	−1.3740	1.4868	2.2086	6.5719

Notes: Market variables use 3752 daily observations from 1 January 2016 to 9 April 2026. Prices are in USD; volume retains the provider’s reported units after K/M/B conversion and is not interpreted as dollar turnover. Change is reported in percentage points. GSADF variables use 3605 initialized observations from 27 May 2016; unavailable pre-initialization flags are not treated as observed zeros. Standard deviations use N−1; skewness and excess kurtosis use bias-corrected sample estimators. The bubble index equals BSADF minus its critical value; the daily flag equals one when the index is positive.

**Table 3:** Candidate models and main parameter settings.

Model	Output Form	Main Parameter Settings
LR	bubble probability	C=1.0; solver = lbfgs; max_iter = 2000; class_weight = balanced
KNN	Neighbor-weighted bubble ratio	n_neighbors = 7; weights = distance; metric=minkowski; p=2
SVM	Calibrated bubble probability	RBF kernel; C=1.0; gamma = scale; probability = True; class_weight = balanced
DT	Leaf-node bubble ratio	max_depth = 5; min_samples_split = 10; min_samples_leaf = 5; class_weight = balanced
RF	Multi-tree mean probability	n_estimators = 300; max_depth = 8; min_samples_split = 5; min_samples_leaf = 2; class_weight = balanced_subsample
XGB	Binary classification probability	n_estimators = 300; max_depth = 4; learning_rate = 0.05; subsample = 0.85; colsample_bytree = 0.85
LightGBM	Binary classification probability	n_estimators = 300; learning_rate = 0.05; num_leaves = 31; max_depth = −1; min_child_samples = 20
ANN	Sigmoid probability	hidden_dims = (128,64); dropout = 0.2; learning_rate = 0.001; epochs = 30
LSTM	Sigmoid probability	hidden_dim = 64; num_layers = 1; dense_dim = 32; dropout = 0.2; learning_rate = 0.001; epochs = 30

**Table 4:** Bitcoin market price-bubble intervals.

Episode	Start Date	End Date	Duration (Days)	Mean Bubble Index	Cumulative Price Change (%)
**Episodes 1–15**					
1	2016-06-03	2016-06-21	19	1.4855	17.7072
2	2016-12-23	2016-12-23	1	0.4096	0.0000
3	2016-12-27	2017-01-04	9	1.5215	22.6399
4	2017-03-02	2017-03-06	5	0.1065	1.7125
5	2017-05-04	2017-05-04	1	0.0141	0.0000
6	2017-05-06	2017-06-30	56	1.7619	60.5255
7	2017-07-02	2017-07-08	7	0.2715	1.1197
8	2017-07-20	2017-07-20	1	0.0540	0.0000
9	2017-08-05	2017-09-12	39	1.4752	27.4641
10	2017-10-08	2017-12-29	83	2.8051	212.0888
11	2017-12-31	2018-01-10	11	0.7529	8.6106
12	2019-05-11	2019-05-16	6	1.2957	9.4780
13	2019-05-19	2019-05-21	3	0.3427	−2.7816
14	2019-05-26	2019-05-29	4	0.5294	0.2039
15	2019-05-31	2019-05-31	1	0.0270	0.0000
**Episodes 16–30**					
16	2019-06-02	2019-06-02	1	0.1094	0.0000
17	2019-06-21	2019-06-29	9	1.0930	16.9678
18	2020-11-17	2020-11-25	9	0.5033	6.0054
19	2020-11-30	2020-11-30	1	0.2168	0.0000
20	2020-12-16	2021-01-26	42	2.4550	52.2190
21	2021-01-28	2021-05-11	104	2.1295	69.8758
22	2023-12-05	2023-12-06	2	0.2698	−0.6804
23	2023-12-08	2023-12-08	1	0.1489	0.0000
24	2024-02-27	2024-03-18	21	1.3052	18.4693
25	2024-03-20	2024-03-21	2	0.2039	−3.4636
26	2024-03-24	2024-04-01	9	0.3899	3.6489
27	2024-04-07	2024-04-08	2	0.1484	3.2723
28	2024-04-10	2024-04-10	1	0.0825	0.0000
29	2024-11-21	2024-11-22	2	0.0688	0.5644
30	2024-12-15	2024-12-17	3	0.1503	1.6238

Notes: An episode is a maximal uninterrupted run of days with BSADF above its critical value (bubble index > 0). All 30 runs are reported, including one-day runs; no duration filter or gap merging is applied. Duration includes both endpoints. Mean bubble index is averaged within each run. Cumulative price change is 100 times (end-date price/start-date price − 1); hence one-day episodes have zero endpoint change. The full observed price sample ends on 9 April 2026. The last detected episode ends on 2024-12-17.

**Table 5:** Horizon-specific sample composition and temporal split.

Horizon	Subset	Origin Period	*N*	Positive	Negative	Positive Share
h=1	Train	2016-06-02 to 2023-04-18	2512	412	2100	16.40%
h=1	Test	2023-04-26 to 2026-04-08	1079	43	1036	3.99%
h=3	Train	2016-06-02 to 2023-04-17	2511	446	2065	17.76%
h=3	Test	2023-04-25 to 2026-04-06	1078	58	1020	5.38%
h=7	Train	2016-06-02 to 2023-04-14	2508	491	2017	19.58%
h=7	Test	2023-04-22 to 2026-04-02	1077	77	1000	7.15%

Notes: Counts refer to the formal revision pipeline with W=7 and a seven-origin purge. The final-test samples are reserved for final evaluation and are not used to select the lookback window, SMOTE setting, or ensemble weights. Origins are dates *t*; the target is one if any daily bubble flag in t+1 through t+h equals one. Train denotes the final development refit sample after purging.

**Table 6:** Development-validation performance across lookback windows.

*W*	Mean Validation PR-AUC	Median Validation PR-AUC	Pooled Validation-Score SD	Rank	Selected
7	0.8521	0.8944	0.1351	1	Yes
10	0.8340	0.8982	0.1594	2	No
14	0.8248	0.8710	0.1670	3	No
30	0.7893	0.8453	0.1938	4	No
60	0.6943	0.6920	0.2452	5	No
90	0.6228	0.6336	0.2888	6	No

Notes: Window selection is performed exclusively on the development period. The three held-out validation periods are 1 August 2017–31 July 2018, 1 January–31 December 2019, and 1 September 2020–31 August 2021. The final test sample is not used for window selection. W=7 is frozen before final evaluation. Without SMOTE, PR-AUC is first averaged across the three validation periods for each model-horizon pair. Mean and median summarize the resulting 27 averages; SD instead summarizes all 81 model-horizon-period scores (sample SD). PR-AUC denotes average precision, not trapezoidal interpolation.

**Table 7:** Selected SMOTE settings and positive-class training counts across forecast horizons.

Model	h=1 SMOTE Ratio	Positive After	h=3 SMOTE Ratio	Positive After	h=7 SMOTE Ratio	Positive After
LR	0.6	1260	0.4	826	0.7	1411
SVM	0.8	1680	0.9	1858	0.9	1815
KNN	0.2	420	Original	446	0.3	605
DT	0.7	1470	0.5	1032	1.0	2017
RF	0.8	1680	0.9	1858	0.7	1411
XGB	1.0	2100	0.8	1652	0.8	1613
LightGBM	0.7	1470	0.9	1858	0.9	1815
ANN	0.6	1260	Original	446	Original	491
LSTM	0.5	1050	0.5	1032	0.5	1008

Notes: All SMOTE settings are selected using development data only and applied only to training windows. Original denotes no oversampling. The ratio is the target minority-to-majority count ratio. Train *N* and class counts before oversampling refer to the original training sample; negative counts are unchanged. Original training samples before oversampling are h=1: N=2512, positive = 412, negative = 2100; h=3: N=2511, positive = 446, negative = 2065; h=7: N=2508, positive = 491, negative = 2017. Forecast horizons are measured in days.

**Table 8:** Development-period rolling pseudo-out-of-sample MSE of base models.

Model	h=1 Mean (SD)	h=3 Mean (SD)	h=7 Mean (SD)
LR	0.0163 (0.0380)	0.0318 (0.0744)	0.0474 (0.1184)
SVM	0.0546 (0.1295)	0.0718 (0.1596)	0.0803 (0.1649)
KNN	0.0345 (0.1155)	0.0462 (0.1304)	0.0644 (0.1623)
DT	0.0228 (0.0602)	0.0231 (0.0533)	0.0464 (0.1110)
RF	0.0147 (0.0370)	0.0208 (0.0520)	0.0278 (0.0715)
XGB	0.0133 (0.0331)	0.0200 (0.0516)	0.0305 (0.0829)
LightGBM	0.0135 (0.0324)	0.0219 (0.0555)	0.0339 (0.0946)
ANN	0.0174 (0.0495)	0.0263 (0.0800)	0.0400 (0.1270)
LSTM	0.0167 (0.0461)	0.0288 (0.0861)	0.0370 (0.1108)

Notes: Values are the mean and sample standard deviation of monthly MSE across 69 development-period rolling pseudo-out-of-sample forecast blocks (ddof = 1). These standard deviations describe temporal variation in forecasting loss and are not standard errors or confidence intervals.

**Table 9:** Final-test performance of 12 models and Naive-0 baseline.

Model	Precision	Recall	F1	PR-AUC	MSE
**Forecast Horizon h=1 Day**					
LR	0.7500	0.7674	0.7586	0.8614	0.0139
SVM	0.8182	0.6279	0.7105	0.8046	0.0171
KNN	0.8065	0.5814	0.6757	0.5360	0.0201
DT	0.5217	0.8372	0.6429	0.7885	0.0272
RF	0.7674	0.7674	0.7674	0.8730	0.0131
XGB	0.7500	0.8372	0.7912	0.8664	0.0138
LightGBM	0.7619	0.7442	0.7529	0.8677	0.0144
ANN	0.8621	0.5814	0.6944	0.7838	0.0178
LSTM	0.8696	0.4651	0.6061	0.7937	0.0204
EW-ME	0.8378	0.7209	0.7750	0.8639	0.0128
IMSE-ME	0.7949	0.7209	0.7561	0.8647	0.0126
CLS-ME	0.7674	0.7674	0.7674	0.8621	0.0125
Naive-0	—	—	—	—	0.0399
**Forecast Horizon h=3 Days**					
LR	0.8298	0.6724	0.7429	0.8439	0.0196
SVM	0.9130	0.3621	0.5185	0.8118	0.0284
KNN	0.9032	0.4828	0.6292	0.5299	0.0285
DT	0.4421	0.7241	0.5490	0.5790	0.0493
RF	0.8649	0.5517	0.6737	0.8315	0.0204
XGB	0.8636	0.6552	0.7451	0.8129	0.0208
LightGBM	0.9024	0.6379	0.7475	0.8601	0.0209
ANN	0.9524	0.3448	0.5063	0.7427	0.0323
LSTM	1.0000	0.3103	0.4737	0.7763	0.0336
EW-ME	0.9375	0.5172	0.6667	0.8150	0.0214
IMSE-ME	0.8919	0.5690	0.6947	0.8108	0.0210
CLS-ME	0.8810	0.6379	0.7400	0.7840	0.0228
Naive-0	—	—	—	—	0.0538
**Forecast Horizon h=7 Days**					
LR	0.9024	0.4805	0.6271	0.7991	0.0311
SVM	1.0000	0.2727	0.4286	0.7319	0.0442
KNN	1.0000	0.4026	0.5741	0.4815	0.0414
DT	0.9070	0.5065	0.6500	0.5233	0.0404
RF	0.9375	0.3896	0.5505	0.7874	0.0354
XGB	0.9024	0.4805	0.6271	0.7280	0.0349
LightGBM	0.9444	0.4416	0.6018	0.7842	0.0382
ANN	0.9583	0.2987	0.4554	0.7120	0.0459
LSTM	1.0000	0.2338	0.3789	0.7469	0.0514
EW-ME	0.9688	0.4026	0.5688	0.8174	0.0362
IMSE-ME	0.9697	0.4156	0.5818	0.8226	0.0361
CLS-ME	0.9211	0.4545	0.6087	0.7993	0.0344
Naive-0	—	—	—	—	0.0715

Notes: Naive-0 assigns probability zero to the bubble state for every final-test observation; therefore, its MSE equals the horizon-specific positive-class share. The final-test sample is not used for window selection, SMOTE selection, ensemble-weight estimation, or main-model selection. Supplementary robustness analyses use the same test period without re-selecting the main specification. PR-AUC denotes average precision. Classification uses probability ≥ 0.5. For Naive-0 only MSE is displayed; dashes denote metrics not reported, rather than all being mathematically undefined.

**Table 10:** Final-test MSE and PR-AUC with 95% block-bootstrap confidence intervals.

Model	MSE [95% CI]	PR-AUC [95% CI]
**Forecast Horizon h=1 Day**		
LR	0.0139 [0.0048, 0.0253]	0.8614 [0.5107, 0.9698]
SVM	0.0171 [0.0074, 0.0285]	0.8046 [0.4067, 0.9421]
KNN	0.0201 [0.0074, 0.0355]	0.5360 [0.1168, 0.7775]
DT	0.0272 [0.0125, 0.0446]	0.7885 [0.4623, 0.9264]
RF	0.0131 [0.0054, 0.0225]	0.8730 [0.5596, 0.9709]
XGB	0.0138 [0.0053, 0.0242]	0.8664 [0.5611, 0.9659]
LightGBM	0.0144 [0.0050, 0.0262]	0.8677 [0.5385, 0.9706]
ANN	0.0178 [0.0064, 0.0318]	0.7838 [0.3661, 0.9364]
LSTM	0.0204 [0.0066, 0.0369]	0.7937 [0.3476, 0.9422]
EW-ME	0.0128 [0.0050, 0.0224]	0.8639 [0.5523, 0.9690]
IMSE-ME	0.0126 [0.0049, 0.0225]	0.8647 [0.5581, 0.9677]
CLS-ME	0.0125 [0.0047, 0.0227]	0.8621 [0.5518, 0.9680]
**Forecast Horizon h=3 Days**		
LR	0.0196 [0.0083, 0.0329]	0.8439 [0.5543, 0.9523]
SVM	0.0284 [0.0115, 0.0481]	0.8118 [0.4870, 0.9470]
KNN	0.0285 [0.0096, 0.0520]	0.5299 [0.0850, 0.8120]
DT	0.0493 [0.0301, 0.0713]	0.5790 [0.2238, 0.7923]
RF	0.0204 [0.0084, 0.0353]	0.8315 [0.5126, 0.9521]
XGB	0.0208 [0.0085, 0.0358]	0.8129 [0.5357, 0.9291]
LightGBM	0.0209 [0.0082, 0.0368]	0.8601 [0.6149, 0.9570]
ANN	0.0323 [0.0119, 0.0558]	0.7427 [0.3887, 0.9210]
LSTM	0.0336 [0.0121, 0.0582]	0.7763 [0.4105, 0.9352]
EW-ME	0.0214 [0.0086, 0.0368]	0.8150 [0.5162, 0.9360]
IMSE-ME	0.0210 [0.0086, 0.0360]	0.8108 [0.5143, 0.9340]
CLS-ME	0.0228 [0.0104, 0.0373]	0.7840 [0.4702, 0.9204]
**Forecast Horizon h=7 Days**		
LR	0.0311 [0.0135, 0.0539]	0.7991 [0.5224, 0.9289]
SVM	0.0442 [0.0193, 0.0747]	0.7319 [0.4083, 0.9135]
KNN	0.0414 [0.0164, 0.0748]	0.4815 [0.1331, 0.7556]
DT	0.0404 [0.0182, 0.0688]	0.5233 [0.2197, 0.7423]
RF	0.0354 [0.0156, 0.0618]	0.7874 [0.4817, 0.9317]
XGB	0.0349 [0.0143, 0.0596]	0.7280 [0.4596, 0.8774]
LightGBM	0.0382 [0.0154, 0.0658]	0.7842 [0.5080, 0.9166]
ANN	0.0459 [0.0192, 0.0777]	0.7120 [0.3806, 0.9166]
LSTM	0.0514 [0.0213, 0.0858]	0.7469 [0.3896, 0.9244]
EW-ME	0.0362 [0.0151, 0.0629]	0.8174 [0.5374, 0.9425]
IMSE-ME	0.0361 [0.0151, 0.0626]	0.8226 [0.5558, 0.9477]
CLS-ME	0.0344 [0.0148, 0.0596]	0.7993 [0.5146, 0.9343]

Notes: Intervals are percentile confidence intervals from 1000 paired moving-block bootstrap replications with block length 10. Resampling is applied jointly to the true label and all 12 frozen model probabilities. The intervals quantify uncertainty conditional on the frozen predictions and do not include model-selection or training uncertainty. PR-AUC denotes average precision. Marginal interval overlap is not a test of differences between models.

**Table 11:** 90% Model Confidence Set results.

Model	h=1 MSE	h=1 MCS *p*	h=3 MSE	h=3 MCS *p*	h=7 MSE	h=7 MCS *p*
LR	0.0139	0.652	0.0196	1.000	0.0311	1.000
SVM	0.0171	0.053 ^†^	0.0284	0.222	0.0442	0.138
KNN	0.0201	0.318	0.0285	0.379	0.0414	0.349
DT	0.0272	0.073 ^†^	0.0493	0.005 ^†^	0.0404	0.114
RF	0.0131	0.684	0.0204	0.945	0.0354	0.343
XGB	0.0138	0.684	0.0208	0.945	0.0349	0.438
LightGBM	0.0144	0.444	0.0209	0.945	0.0382	0.329
ANN	0.0178	0.297	0.0323	0.369	0.0459	0.240
LSTM	0.0204	0.417	0.0336	0.368	0.0514	0.193
EW-ME	0.0128	0.684	0.0214	0.683	0.0362	0.349
IMSE-ME	0.0126	0.714	0.0210	0.945	0.0361	0.349
CLS-ME	0.0125	1.000	0.0228	0.222	0.0344	0.438

Notes: The 90% MCS uses squared-error loss, size 0.10, 1000 stationary-bootstrap replications, expected block size 10, method R, and seed 42. A dagger (^†^) denotes exclusion (MCS *p*-value < 0.10). Retention does not establish equivalence or superiority. Only conventional MCS membership is interpreted.

**Table 12:** LSTM robustness with and without SMOTE.

Horizon	Setting	MSE	PR-AUC	Recall	Precision	F1
h=1	SMOTE	0.0204	0.7937	0.4651	0.8696	0.6061
h=1	No SMOTE	0.0187	0.8290	0.5116	0.9167	0.6567
h=3	SMOTE	0.0336	0.7763	0.3103	1.0000	0.4737
h=3	No SMOTE	0.0345	0.8094	0.2759	1.0000	0.4324
h=7	SMOTE	0.0514	0.7469	0.2338	1.0000	0.3789
h=7	No SMOTE	0.0524	0.7601	0.2078	1.0000	0.3441

Notes: All LSTM hyperparameters, temporal splits, thresholds, and random-seed policy are held fixed; only the use of SMOTE changes. SMOTE is applied only to training windows. The comparison is a robustness check and is not used to re-select the formal main model. PR-AUC denotes average precision; the probability classification threshold is 0.5.

**Figure A2:**
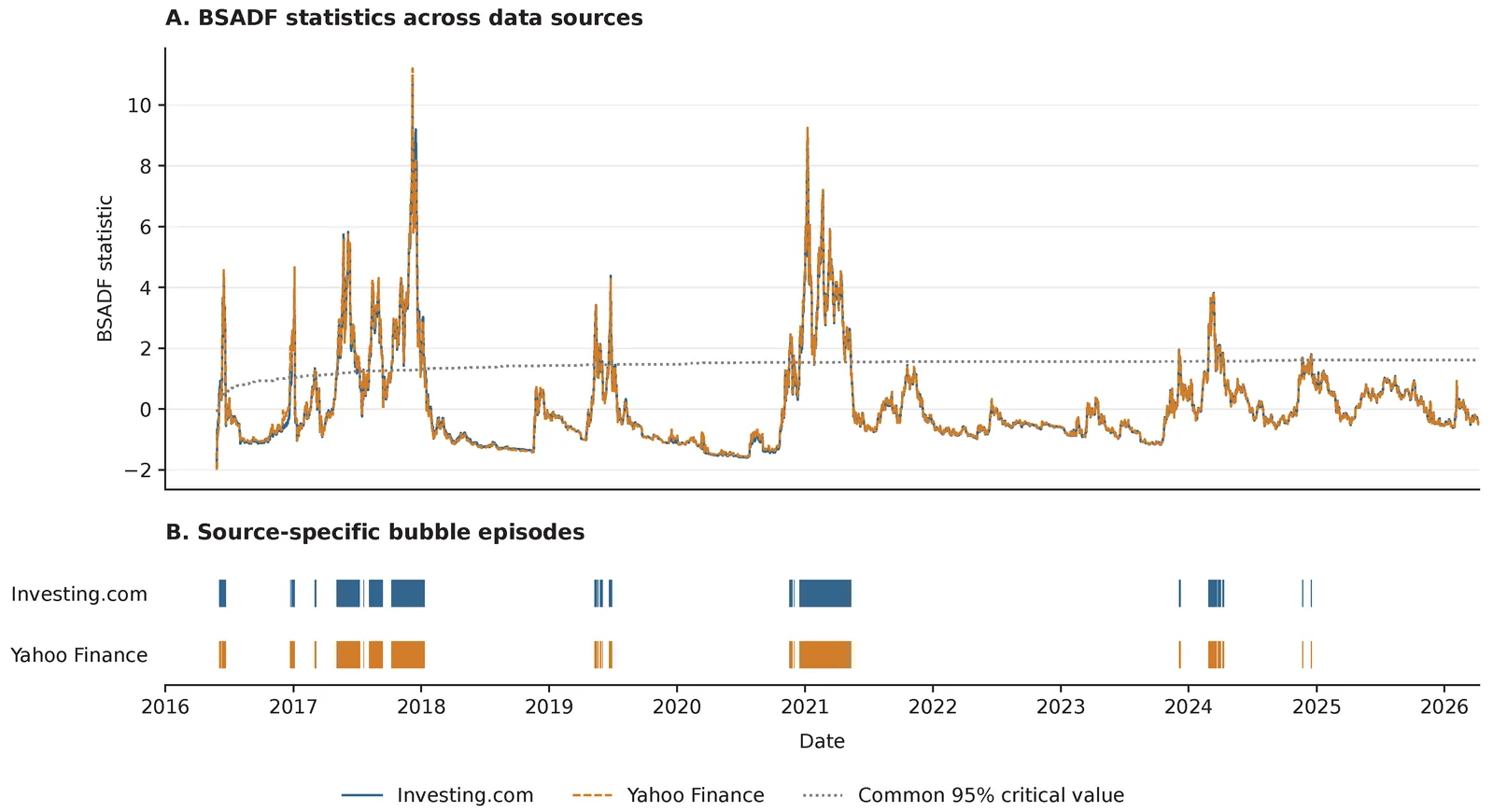
Cross-source comparison of GSADF-based Bitcoin bubble periods. The upper panel compares the BSADF sequences and their common 95% critical value; the lower panel shows each provider’s maximal uninterrupted bubble intervals. Both series use the same sample and specification; each shaded interval has its actual calendar width.

## Data Availability

The primary raw data analyzed in this study are available from Investing.com. Yahoo Finance BTC/USD prices are used only for the cross-source robustness analysis. The processed data supporting the findings of this study are available from the corresponding author upon reasonable request.

## Appendix A. Validation Sensitivity and Class-Weight Ablation

Figure A1 and Table A1 report validation sensitivity and class-weight ablation results.

Table A1 is restricted to LR, SVM, DT, and RF because their main specifications include class weighting.

**Table A1:** SMOTE × class-weight ablation.

Model	Setting	MSE	PR-AUC	Recall	Precision	F1
**Forecast Horizon h=1 Day**						
LR	A	0.0140	0.8566	0.6977	0.7895	0.7407
LR	B	0.0139	0.8617	0.6977	0.7500	0.7229
LR	C	0.0142	0.8597	0.7674	0.7500	0.7586
LR	D	0.0139	0.8614	0.7674	0.7500	0.7586
SVM	A	0.0194	0.7600	0.4651	0.8696	0.6061
SVM	B	0.0175	0.7957	0.6279	0.8438	0.7200
SVM	C	0.0206	0.7639	0.4186	0.9000	0.5714
SVM	D	0.0171	0.8046	0.6279	0.8182	0.7105
DT	A	0.0157	0.6349	0.8837	0.7308	0.8000
DT	B	0.0240	0.7240	0.8605	0.5441	0.6667
DT	C	0.0304	0.7548	0.8372	0.5294	0.6486
DT	D	0.0272	0.7885	0.8372	0.5217	0.6429
RF	A	0.0128	0.8674	0.7674	0.7857	0.7765
RF	B	0.0131	0.8670	0.7674	0.7674	0.7674
RF	C	0.0134	0.8638	0.7907	0.7391	0.7640
RF	D	0.0131	0.8730	0.7674	0.7674	0.7674
**Forecast Horizon h=3 Days**						
LR	A	0.0209	0.8539	0.5862	0.8718	0.7010
LR	B	0.0200	0.8474	0.6034	0.8537	0.7071
LR	C	0.0190	0.8522	0.6379	0.8222	0.7184
LR	D	0.0196	0.8439	0.6724	0.8298	0.7429
SVM	A	0.0283	0.6958	0.4483	0.9286	0.6047
SVM	B	0.0275	0.8020	0.3966	0.8846	0.5476
SVM	C	0.0333	0.7179	0.2931	1.0000	0.4533
SVM	D	0.0284	0.8118	0.3621	0.9130	0.5185
DT	A	0.0291	0.6530	0.6724	0.6964	0.6842
DT	B	0.0384	0.7156	0.7759	0.4688	0.5844
DT	C	0.0439	0.6781	0.7241	0.6562	0.6885
DT	D	0.0493	0.5790	0.7241	0.4421	0.5490
RF	A	0.0188	0.8419	0.6552	0.8837	0.7525
RF	B	0.0200	0.8357	0.5690	0.8462	0.6804
RF	C	0.0199	0.8354	0.5690	0.8462	0.6804
RF	D	0.0204	0.8315	0.5517	0.8649	0.6737
**Forecast Horizon h=7 Days**						
LR	A	0.0345	0.8140	0.4156	0.9143	0.5714
LR	B	0.0321	0.8012	0.4675	0.9000	0.6154
LR	C	0.0298	0.8081	0.5195	0.8889	0.6557
LR	D	0.0311	0.7991	0.4805	0.9024	0.6271
SVM	A	0.0422	0.6031	0.3766	0.9355	0.5370
SVM	B	0.0435	0.7317	0.2727	1.0000	0.4286
SVM	C	0.0499	0.6303	0.1818	1.0000	0.3077
SVM	D	0.0442	0.7319	0.2727	1.0000	0.4286
DT	A	0.0368	0.5244	0.6104	0.8103	0.6963
DT	B	0.0404	0.5233	0.5065	0.9070	0.6500
DT	C	0.0316	0.6265	0.6753	0.7879	0.7273
DT	D	0.0404	0.5233	0.5065	0.9070	0.6500
RF	A	0.0321	0.8014	0.4675	0.8780	0.6102
RF	B	0.0348	0.7924	0.3896	0.9375	0.5505
RF	C	0.0351	0.8005	0.4026	0.9118	0.5586
RF	D	0.0354	0.7874	0.3896	0.9375	0.5505

Notes: A: neither SMOTE nor class weighting; B: SMOTE only; C: class weighting only; D: simultaneous SMOTE and class weighting (official main specification). All non-factor settings and temporal splits are held fixed. The ablation is supplementary and does not re-select the formal main configuration. PR-AUC denotes average precision; the probability classification threshold is 0.5.

## Appendix B. Cross-Source GSADF Robustness

To assess the sensitivity of GSADF bubble dating to the price-data provider, the main procedure is repeated using Yahoo Finance BTC/USD daily prices over the identical 1 January 2016–9 April 2026 sample. The Yahoo series contains the same 3752 calendar-day observations and is analyzed with the same lag order (0), minimum window (147), price-level specification, and 95% critical-value sequence as the primary Investing.com series. No interpolation, temporal shifting, or gap merging is applied.

Table A2 summarizes agreement over the 3605 initialized daily observations. Figure A2 compares the two BSADF sequences and the dated bubble intervals.

**Table A2:** Cross-source robustness of GSADF-based Bitcoin bubble identification.

Measure	Investing.com	Yahoo Finance	Comparison
Price observations	3752	3752	Identical
Initialized observations	3605	3605	Identical
GSADF statistic	10.6858	11.2011	+4.82%
Bubble days	455	454	−1
Bubble episodes	30	30	0
Daily state agreement	—	—	0.9958
Bubble-day intersection	—	—	447
Bubble-day union	—	—	462
Jaccard similarity	—	—	0.9675
Investing-day coverage by Yahoo	—	—	0.9824
Yahoo-day coverage by Investing	—	—	0.9846
BSADF Pearson correlation	—	—	0.9983
BSADF Spearman correlation	—	—	0.9978

Notes: Both price series cover 1 January 2016–9 April 2026. Daily state agreement and correlations use only initialized observations. Jaccard similarity equals intersection divided by union. Directional coverage divides the intersection by each provider’s bubble-day count. The first 147 observations are unavailable for bubble classification, not classified as non-bubble days.
